# Oral appliance therapy in obstructive sleep apnea: Long-term adherence and patients’ experiences

**DOI:** 10.4317/medoral.22158

**Published:** 2017-12-24

**Authors:** Banu Saglam-Aydinatay, Tülin Taner

**Affiliations:** 1 Assistant Professor, Department of Orthodontics, Faculty of Dentistry, Hacettepe University, Ankara, Turkey; 2 Professor, Department of Orthodontics, Faculty of Dentistry, Hacettepe University, Ankara, Turkey

## Abstract

**Background:**

Despite the advances in the treatment of obstructive sleep apnea (OSA) with mandibular advancement appliances (MAA), their effectiveness is dependent on the patients’ compliance. Our aims were to evaluate the long-term adherence to MAA therapy and patients’ experiences of the treatment in OSA.

**Material and Methods:**

Sixty-nine patients (52 males, 17 females; Mean age: 54.4±10.8 years) were included in the study. The subjects were mild (56%) and moderate (44%) OSA patients who had been treated using MAA at least 4 years prior to the study. A phone survey was used to determine the demographic characteristics of the patients, as well as to assess self-reported adherence to therapy, subjective long-term effectiveness, and patient experiences with the appliance. Descriptive statistics, Pearson Chi-square test, and independent samples t-test were used for data analysis.

**Results:**

Only 22 (32%) patients reported using the appliance regularly. Most of the non-adherent patients had stopped using their appliances in the first year (55%). The mean duration of appliance use was 33.5 months (Median: 12 months). No significant differences in appliance type, OSA severity, educational level, gender, marital status, income status, employment status or place of residence existed between adherent and non-adherent subjects. Adherent subjects were significantly younger than non-adherent subjects (Age: 50.6 ± 11.9 versus 56.1 ± 9.9, *p*<0.05). The most common reasons reported by patients were inability to adapt to the appliance (62%) and pain in the temporomandibular joint (38%). The most common factors associated with continued usage were effectiveness (100%) and ease of use (64%).

**Conclusions:**

The overall long-term nonadherence to MAA therapy in mild-to moderate OSA patients was high suggesting that barriers to MAA therapy adherence should be prevented to increase the efficiency of oral appliance treatment in OSA and achieve better outcomes for this disease.

** Key words:**Obstructive sleep apnea, mandibular advancement, therapy, oral appliance, long-term compliance.

## Introduction

Obstructive sleep apnea (OSA) is a highly prevalent sleep-related breathing disorder characterized by periods of recurrent cessation of breathing caused by partial or complete collapse of the upper airway. Untreated OSA is associated with cardiovascular disorders, cerebrovascular disease, and cognitive dysfunction (1). The gold standard treatment for OSA is continuous positive airway pressure (CPAP) therapy to prevent upper airway collapse during sleep. However, adherence to CPAP is often poor, which limits its efficacy (2). Other treatment alternatives include oral appliances (OA), various surgeries and/or adjunctive measures such as weight loss (1).

Mandibular advancement appliances (MAA) act by protruding the mandible and increasing the upper airway size and they are the most commonly used type of OA’s in the treatment of OSA (3). Their use is indicated in patients with mild to moderate OSA, and in individuals who are intolerant to CPAP treatment (4). They have been shown to be effective in reducing snoring and obstructive breathing events as well as improving health outcomes in the short-term (5). Long-term studies have also reported continuing effectiveness in terms of Apnea-Hypopnea Index (AHI) and Oxygen Desaturation Index (ODI) up to 5 years (6-8), while others have found that AHI increases with time (9,10). Comparative studies show that most patients prefer oral appliances over CPAP (5,11,12).

Despite the advances in the treatment of sleep apnea with oral appliances, their effectiveness is dependent on the patients’ adherence. Adherence to a medical regimen is defined as the successful adoption of a treatment program (13). According to the World Health Organization (WHO), 50% of patients with chronic diseases do not follow treatment recommendations (14). Long-term compliance studies with oral appliances ranging in follow-up durations from 1 to 10 years report a wide range of adherence between 4-90% (8,15). Therefore, our aims in this study were to evaluate the long-term adherence rates to MAA therapy and patients’ reported experiences of the treatment.

## Material and Methods

After obtaining the institutional ethics committee approval, 77 patients (60 males, 17 females) who were successfully treated with an MAA at our clinic between 2005 and 2012 (at least 4 years prior to the study) were contacted by phone. These patients had been diagnosed with sleep apnea at the Department of Otorhinolaryngology or the Department of Chest Diseases using an overnight polysomnography. Their disease severity had been classified as mild (AHI ≥ 5 and < 15, n=46) or moderate (AHI ≥ 15 and < 30, n=31) (16), and they have been referred to our clinic for treatment with an OA. Two different appliance designs had been used in the treatment of patients. One group (n=52) had been fitted with the monobloc appliance, and the second group (n=25) with a modified twin-block appliance (17). Efficacy of the oral appliance had been determined by an overnight polysomnography during which the patients wore their appliances. The treatment had been considered successful if there was a reduction in AHI to < 5 events per hour with a > 50% resuction in baseline AHI.

A phone survey was used to determine the demographic characteristics of the patients, as well as to assess self-reported adherence to therapy, subjective long-term effectiveness, and patient experiences with the appliance. Patients who stopped using their appliances were classified as ‘’non-adherent’’, while those who were still using their MAA’s were classified as ‘’adherent’’.

-Statistical analysis

Statistical analyses were performed using SPSS, Version 21.0 (IBM SPSS Statistics for Windows, Version 21.0. Armonk, NY: IBM Corp.). Demographic data were analyzed using descriptive statistics which included the mean, standard deviation and percentage distribution. Categorical variables were compared between adherent and non-adherent groups using Pearson Chi-square test. For continuous variables independent samples t-test was used. Statistical significance was defined as *p* < .05 (two-tailed).

## Results

Demographic characteristics of the patients are presented in  Table 1. Out of the initial 77 patients, we were able to reach 69 (90%) by phone. All of the patients verbally consented to answer the questionnaire. The patients included in the study were treated with either a Monobloc (n=47 (68%)) or a Modified Twin-block Appliance (n=22 (32%)). The responding group consisted of 39 patients (56%) with mild OSA and 30 patients (44%) with moderate OSA. The mean age of the sample was 54.4±10.8 years (54±11.4 years in males; 55.6±8.2 years in females), with the range of ages varying from 29 years to 76 years old. Most of the patients were male (75%), married (96%), currently employed (56%), lived in an urban area (94%), had an educational level of high school or higher (83%), and reported medium income (59%). The majority of respondents commented that their appliance was successful in decreasing their symptoms (74%), comfortable to use (58%), and easy to maintain (88%). Seventy per cent of those interviewed replied that they would recommend oral appliance therapy to their friends. ‘’Difficulty of use’’ was the most commonly cited reason (n=12; 60%) by those who wouldn’t recommend the treatment, followed by ‘’ineffectiveness’’ (n=7; 35%).

**Table 1:**
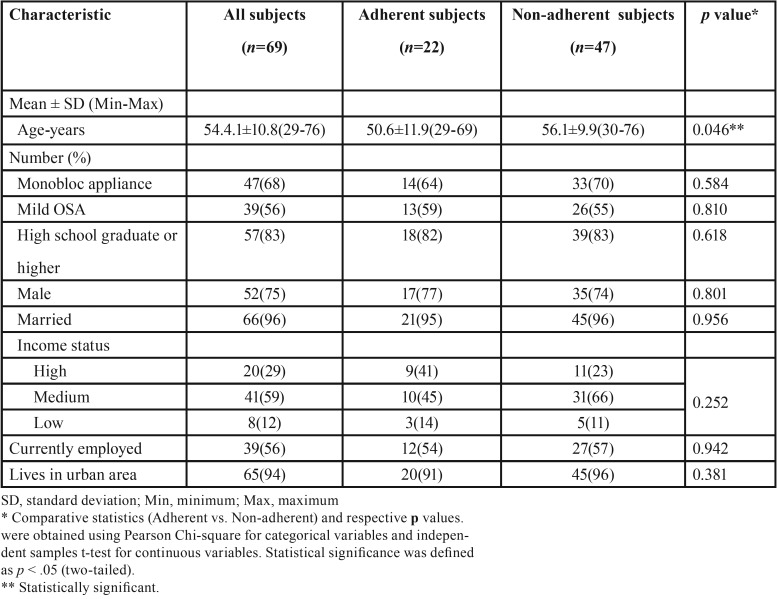
Demographic characteristics of the patients.

**Figure 1:**
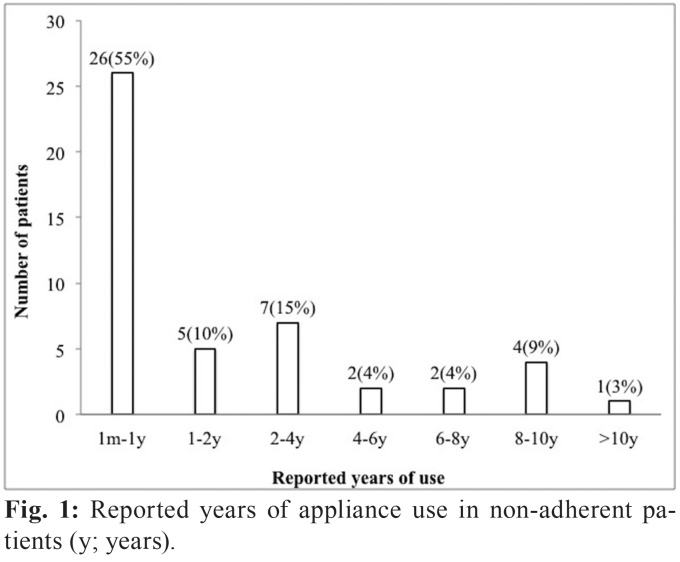
Reported years of appliance use in non-adherent patients (y; years).

Only 22 (32%) subjects reported using the appliance regularly. Most of the non-adherent patients had stopped using their appliances in the first year (55%), 10% in the second year and another 15% in the fourth year (Fig. 1). The mean duration of appliance use was 33.5 months (Median: 12 months; Range: 1-132 months). No significant differences in appliance type, OSA severity, educational level, gender, marital status, income status, employment status or place of residence existed between adherent and non-adherent subjects. Adherent subjects were significantly younger than non-adherent subjects (Age: 50.6 ± 11.9 versus 56.1 ± 9.9, *p* < .05).

The reasons given for stopping appliance use are shown in  Table 2. The most common reasons reported by patients were inability to adapt to the appliance (62%), pain in the temporomandibular joint (38%), ineffectiveness in decreasing symptoms (28%), and dry mouth (28%). As shown in  Table 3, the factors associated with continued usage were effectiveness (100%), ease of use (64%), support from their partner (32%), the shame caused by disease symptoms (32%), and portability of the appliance (27%).

**Table 2:**
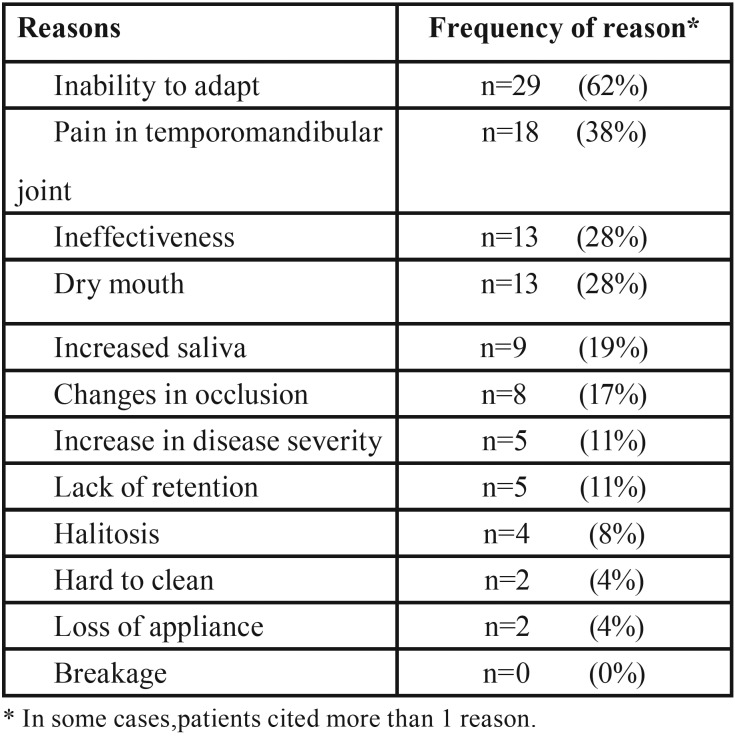
Self-reported reasons for stopping appliance use.

**Table 3:**
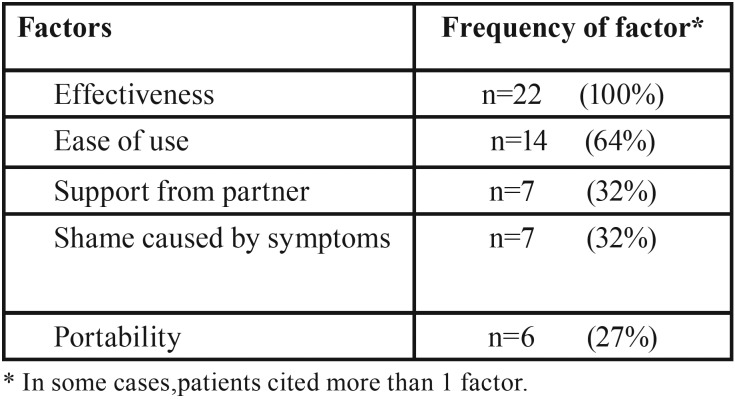
Self-reported factors associated with continued usage.

Only 38% of the non-adherent subjects seeked other treatments for their OSA. Of these patients, 50% were using CPAP and 31% had undergone upper airway surgery ( Table 4).

**Table 4:**
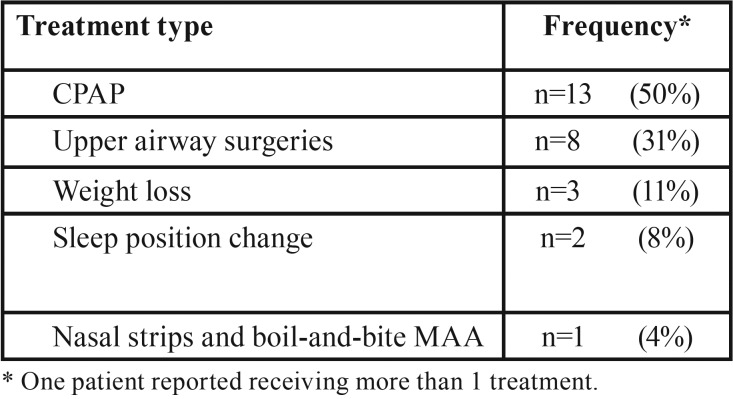
Treatment types in non-adherent patients who received further treatments (n=26).

## Discussion

This study aimed to determine adherence to treatment and long-term MAA experience in a group of patients in a university clinic. A phone survey was preferred because the response rate of telephone follow-up is reported to be higher (18). We were unable to contact 8 patients, possibly due to changes in phone number or an unwillingness to answer a call from an unknown number. However, all the patients that we were able to reach consented to answer the survey which resulted in a high response rate.

Lack of adherence to therapy is a well-recognized problem in chronic conditions and results in potentially avoidable health risks as well as unnecessary health care spending (19). Studies show that adherence rates in MAA treatment are also suboptimal. In a systematic review, Hoffstein (15) reported a wide range of (4-76%) compliance rates in the first year of appliance use. Other studies found that adherence decreased with time: 83% after 1 year (20), and 62-64% after 4-6 years (10,21). Our findings demonstrated an adherence rate of 32% in long-term (4-11 years) MAA therapy of mild-to-moderate OSA patients. Adherence in the current study was also lower than the 60% compliance rate reported in a 10-year study by Wiman Eriksson *et al.* (8). Access to healthcare, cultural beliefs, education about chronic disease, and the nature of patient-physician interactions may vary between countries (22) which could have been a contributing factor to the low compliance rate in our study.

However, even the patients who were not using their appliances were willing to recommend MAA therapy to a friend, which is in aggreement with the findings of Nordin *et al.* (23). Consistent with the results of de Almeida *et al.* (21), we found that the highest percentage of dropouts (55%) occurred in the first year of appliance use. This pattern of withdrawal from therapy can also be found in CPAP adherence studies (24). The major reasons for discontinuation of MAA therapy in our study were inability to adapt to the appliance, pain in the temporomandibular joint, ineffectiveness in decreasing symptoms, and dry mouth. Similarly, excessive salivation, xerostomia, tooth and gingival discomfort, and self-appreciated lack of efficacy are the most common side effects reported in literature (6,15,21,23). There were also facilitators associated with continued usage such as effectiveness, ease of use, support from their partner, the shame caused by disease symptoms, and portability of the appliance. These results emphasize the need for a good communication between the clinician, the patient and their family. The patients’ complaints should be adressed by the dentist, and they should be informed that the side effects are temporary in many cases (15). Furthermore, incorporating educational, technological, psychosocial or multi-dimensional strategies that have been tested in CPAP adherence may also help in increasing the long-term adherence rates in oral appliance therapy (25).

Most of the patients in our study (68%) were treated with a monobloc appliance, which was the most frequent type of MAA at the time. Some studies report that monobloc appliances are as effective as bibloc appliances although the effect remains uncertain (26). Patient satisfaction has also been found to be comparable for these different appliance types (23). Dieltjens *et al.* (27) found that MAA type was a predictor of treatment discontinuation, with an odds ratio of 9.12 with monobloc MAA leading to a higher discontinuation rate compared to bibloc appliances. In contrast with these results, we found no significant differences in appliance type between groups. There were also no differences in OSA severity, educational level, gender, marital status, income status, employment status or place of residence. The only predictor for adherence in our study was age. The effect of age on oral appliance adherence is controversial. Carballo *et al.* (28) found that adherence among older adults was lower, whereas Dieltjens et al. (27) have noted no association between these two variables. We found that adherent subjects were significantly younger than non-adherent subjects. One possible reason for these conflicting results is the differences in age groups between the study populations. Another explanation could be that any reduced adherence noted as a function of advancing age may be mediated by other factors such as changes in the dentition or the mucosal tissue making it harder for older patients to adapt to oral appliances. Further studies with larger samples that include all age groups is needed for better understanding the role of age in oral appliance therapy.

Almeida *et al.* (21) reported that 23% of moderate-to-severe OSA patients non-adherent to MAA therapy use CPAP after stopping oral appliance use. Similarly, McGown *et al.* (29) found that 14% of patients choose to use CPAP after trying MAA therapy. Consistent with these previous studies, we determined that 50% of the non-adherent subjects who seeked other treatments for their OSA were using CPAP.

Our research has some limitations. The first one is the use of subjective compliance data to determine adherence rates. Despite the limitations of this method, it is still commonly used in adherence studies. Furthermore, a high agreement between objective and subjective compliance data in oral appliance therapy has been reported in literature (16). Nevertheless, social desirability response bias and recall bias cannot be totally ruled out, especially in long-term survey studies. Another major limitation is the sample size of the group. Since this was a single-center study with long-term follow-up we failed to include a higher number of patients. Thus, future research would benefit from multi-center studies with larger sample sizes to increase the generalizability of the findings and objective compliance data which can be gathered by using the newly developed compliance monitors embedded within the appliances (30).

In conclusion, long-term adherence rates in MAA therapy of mild-to-moderate OSA patients is lower than those reported in short-term studies and younger patients show higher compliance rates. Since oral appliance therapy is the first-line of treatment for a number of these patients, factors affecting adherence should be explored in future studies. Furthermore, interventions aimed at improving adherence should be designed to ensure the successful treatment of this chronic disease.
